# Roughness and SEM Analysis of Manual and Ultrasonic Instrumentation over Different Crown Materials for Dental Implants Restorations

**DOI:** 10.3390/ma15031159

**Published:** 2022-02-02

**Authors:** Domenico Baldi, Jacopo Colombo, Paola Gavoglio, Luisa De Giorgis, Franco Motta, Andrea Lugas, Enrico Lertora, Gianmario Schierano

**Affiliations:** 1 Division of Implant Prosthodontics, Department of Surgical Sciences, University of Genova, Via Piacenza 86, 16138 Genova, Italy; baldi.domenico@libero.it (P.G.); ludeg94@gmail.com (L.D.G.); motta.studio@tiscali.it (F.M.); 2 Private Practice, 19100 La Spezia, Italy; jacopocolo@tiscali.it; 3 Solid and Fluid Biomechanics Group, PolitoBIOMed Lab, Department of Mechanical and Aerospace Engineering, Turin Polytechnic School, 10129 Turin, Italy; jacopocolo@hotmail.it; 4 Department of Mechanical Engineering, Polytechnic School, University of Genova, 10129 Turin, Italy; lertora.enrico@unige.it; 5 Department of Surgical Science, C.I.R. Dental School, University of Turin, 10124 Torino, Italy; gianmario.schierano@unito.it

**Keywords:** implant dentistry, implant prosthodontics, prosthetic materials, dental hygiene

## Abstract

The use of new prosthetic materials makes it necessary to establish adequate hygienic protocols. It was decided to make prosthetic crowns from four different materials: composite, lithium disilicate, metal ceramic, and zirconium, and to evaluate the effects on the surfaces of four different instruments through SEM and roughness analysis: manual steel curette, manual titanium curette, ultrasonic steel insert, and ultrasonic peek insert. Forty crowns were made, ten of each type of material. For each material, five crowns were manually instrumented with steel inserts (curette 11-12, PDT, Missoula, MT, USA) and titanium (Wingrove 3-4, PDT, Missoula, MT, USA) on the lingual and buccal surfaces, respectively, and the other five crowns were instrumented with an ultrasonic peek insert (ICS-IC1, Mectron, Carasco, Italy) on the buccal surface and steel (PS, EMS, Nyon, Switzerland) on the lingual surface. At this point, surface roughness analysis was carried out. The data were analyzed with a Kolmogorov–Smirnov test. Therefore, it was decided to conduct two analyses with a Kruskal–Wallis test and Bonferroni post hoc test. Then, the instrumented crowns were analyzed by SEM. The analysis of the data shows that the highest average roughness was within the composite group, while the best material appeared to be disilicate. Significant differences existed between the groups, between the materials, and between the different instruments (*p*-value < 0.05). In the qualitative analysis carried out by SEM, the classic steel insert eliminated the residues of golden finishing. The peek insert created alterations on all tested surfaces. The steel curette did not create particular problems, with the exception of zirconium, where it was possible to observe some scratch lines. Instrumentation with the titanium curette created deeper incisions than the steel curette in the composite and disilicate. The best results came from the ultrasonic steel insert, while the best material appeared to be disilicate.

## 1. Introduction

In recent years, as established by the WHO (World Health Organization) in a report published in 2009, due to socio-economic changes, there has been a constant increase in the average life span of the population [1]. This, accompanied by the growing aesthetic needs of patients, has contributed to an increase in the demand for fixed dental prostheses in all aspects: single crowns, bridges, veneers, and inlays with anchoring on natural teeth and especially on implants [2]. The aim of prosthetic therapy is to restore the shape and physiological function of the dentition [3]. The prosthesis should be as simple and conservative as possible, but be able to satisfy specific functional, phonetic, aesthetic, and mechanical requirements [4,5]. Fixed prosthesis is a solution and is best accepted by the patient [6]. The complete dental hygiene of the oral cavity involves the care of all natural teeth, soft tissues, and dental prostheses and any supporting implants. Patients with prostheses are more prone to tooth decay and periodontal infections due to the accumulation of biofilm present at the margins of the restorations and under the pontic elements [7]. 

It is now necessary to know the type and materials from which the prostheses are manufactured, the support tissues, and the most common problems that a patient may have with the prostheses themselves in order to provide information, comprehensive instructions, and suitable treatments. Proper oral hygiene and daily maintenance by the patient are essential factors for the success and longevity of a prosthesis and for the health of the remaining teeth and oral tissues. We can understand how important it is to create an educational program of professional and home maintenance for each patient according to individual needs and based on the type of prosthesis [8].

Removable prostheses are removed from the oral cavity before the operating hygiene session and, where necessary, treated with manual or ultrasonic instruments outside the oral cavity.

Fixed prostheses, on the other hand, as they cannot be removed, form a whole structure with natural teeth or implants, and they must be instrumented within the oral cavity.

For the manual instrumentation of the implant surfaces, curettes in synthetic material (carbon fiber or hard plastic) and inserts in plastic material (peek) or carbon composite are used for ultrasonic instrumentation in order to avoid alterations of the implant surfaces [9,10]. For the treatment of fixed prostheses, there are no validated protocols for maintenance sessions, even if we tend to use manual instruments, such as curettes, or mechanical instruments, such as ultrasounds, knowing the characteristics of their functioning that have made them extremely useful in other dental practices [11,12,13,14,15].

In clinical practice, we are faced with aesthetic crowns, and these, like implants, must not be subjected to alterations of their surface. This is important both in order not to compromise the chemical–physical characteristics of the material and to avoid an accumulation of biofilm, which, as is known, is favored by rough and irregular surfaces. Alterations of the surfaces of prostheses and therefore roughness are also unsightly. The purpose of this study is to evaluate the effects of manual and ultrasonic instrumentation on crowns made from different aesthetic materials used in dentistry today.

## 2. Materials and Methods

Ten crowns were made for each material to be tested: composite, lithium disilicate, ceramic, and zirconium. All of the most current and most widely used laboratory procedures in the dental technician field were followed for the realization of the prosthetic products, with the aim of borrowing the widest range of possible clinical situations found in the oral cavity. The composite crowns were made using the reverse layering technique (IPS Empress Direct, Ivoclar Vivadent, Naturno, Italy). The construction of the lithium disilicate crowns was carried out using the CAD (computer-aided design)–CAM (computer-aided manufacturing) technique with a software-assisted technical drawing and milling of the crown starting from a pre-finished block (IPS E. Max, IvoclarVivadent, Naturno, Italy). The metal ceramic crowns were made from a fused metal structure (chrome–cobalt alloy) that supports the layering of the aesthetic ceramic (IPS E.Max Ceram, IvoclarVivadent, Naturno, Italy). For the zirconium crowns, we proceeded with the CAD–CAM technique to obtain a substructure, which was then veneered with the layering technique (IPS E.Max ZirCad, IvocalrVivadent, Naturno, Italy).

The crowns were made on implant abutments and a didactic model was used as a support for the crowns both in the manufacturing phase and during the study phases (Kavo, Biberach, Germany). For SEM analysis, the sample was made conductive in its surface layer by covering it with a thin layer of gold (coating or metallization).

The samples were then covered with an 8-nanometer layer of gold using a metallizer (Polaron Sputter Coater SC7640, Ladd, Williston, VT, USA) and SEM analysis was performed (Zeiss EVO 40, Carl Zeiss AG, Oberkochen, Germany).

Subsequently, the samples were instrumented with various instruments, in particular:

- All the vestibular surfaces of the first five elements of each material were mechanically treated with a peek insert (ICS-IC1, Mectron, Carasco, Italy) mounted on a MultiPiezo ultrasonic motor (Mectron, Carasco, Italy);
- All the lingual surfaces of the first five elements of each material were mechanically treated with a steel insert (PS, EMS, Nyon, Switzerland) mounted on a Mini Piezon ultrasonic motor (EMS, Nyon, Switzerland);
- All the vestibular surfaces of the second five elements of each material were manually treated with titanium curettes (Wingrove 3-4, PDT, Missoula, MT, USA);
- All the lingual surfaces of the second five elements of each material were manually treated with 11-12 steel curettes (PDT, Missoula, MT, USA).

In collaboration with the Department of Materials Engineering of Genoa University, the surface roughness was evaluated. The roughness was assessed using a roughness tester for mechanics (Mitutoyo SJ-301 Surf Test, Mitutoyo Corp, Kawasaki, Japan). This instrument was provided with a sensor constituted by a head with a diamond tip of 2 μm in size. The sensor transmits data to a central data-processing body through an inductive detection system. The processing body can collect and analyze twenty measurements at a time. The engineer measuring the dental roughness was blinded to the type of instrument used. Then, the samples were re-browned and analyzed by SEM after instrumentation.

## 3. Statistical Analyses

The data obtained were subjected to a Kolmogorov–Smirnov normality test to decide which test to perform to evaluate the significance of the differences. Since the data were not normally distributed, the analysis continued with the Kruskal–Wallis test. Two Kruskal–Wallis analyses were carried out to evaluate the influence of the material and instrumentation on the surface roughness, considering the average surface roughness Sa as an indicative parameter. To determine between which materials and equipment there was a significant difference, a Bonferroni post hoc test was performed.

## 4. Results

In the first part of this section, the characteristics detected by the SEM of the non-instrumented aesthetic surfaces will be described. Later, we will deepen the characteristics of the surfaces themselves following instrumentation.

1. Composite

**Figure 1:**
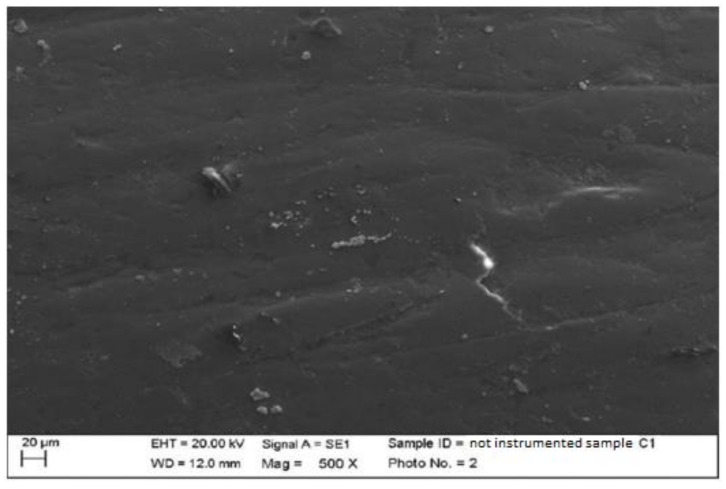
Non-instrumented composite sample C1 (500×).

**Figure 2:**
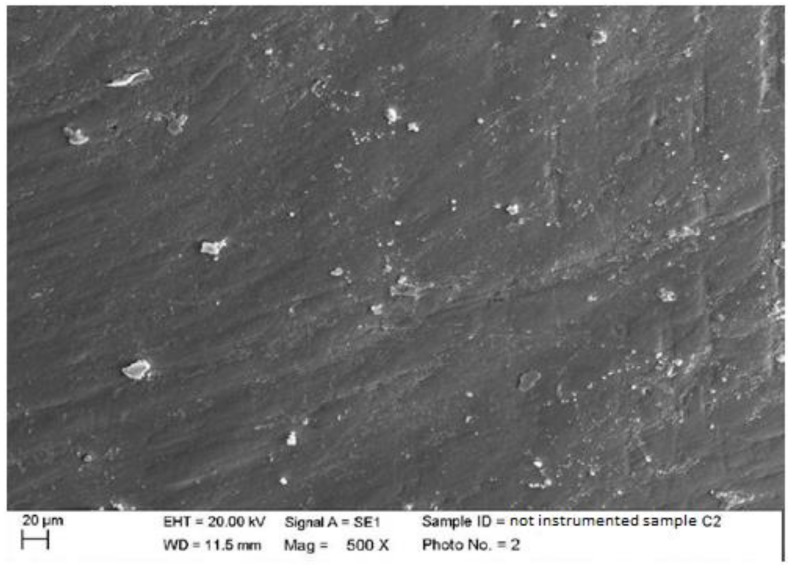
Non-instrumented composite sample C2 (500×).

There were processing residues on the composite, but to a lesser extent than on the ceramic, as can be seen in the following images. Some streaks and concavities with clear edges that represent the irregularity of the surface of the prosthetic element were clearly visible in the highest magnifications (500×, 2000×) (Figure 1). In the second sample, it is possible to notice at the 500× magnification the presence of slight surface incisions caused by the tools used for the final polishing of the composite element (Figure 2).

2. Lithium disilicate

**Figure 3:**
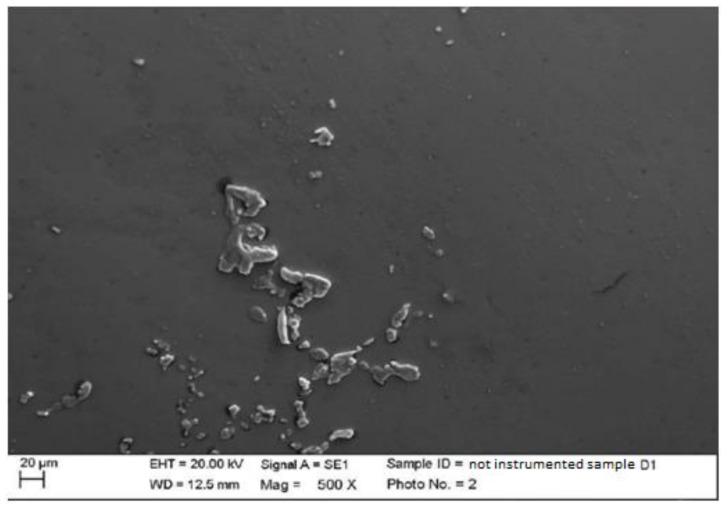
Non-instrumented disilicate sample D1 (500×).

The surface of the lithium disilicate had a very smooth appearance. Processing residues, especially subsurface, were present, but in smaller quantities than the other materials; however, the dimensions of the individual particles were larger (Figure 3).

3. Ceramic

**Figure 4:**
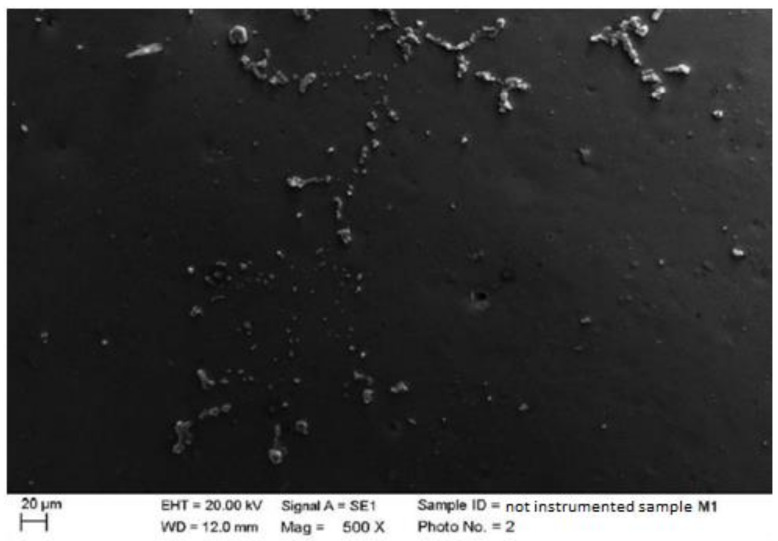
Non-instrumented ceramic sample M1 (500×).

As for the elements made of ceramic material with a metal substructure, we could see, in an increasingly defined manner as the magnification increased (500×), residues from the processing of the material itself. In particular, the residues incorporated within the structure of the material were visible to the maximum degree (2000×) as if they were covered by a thin film. There were also small depressions, which confirmed that the ceramic material was a hard but, at the same time, fragile material (Figure 4).

4. Zirconium

**Figure 5:**
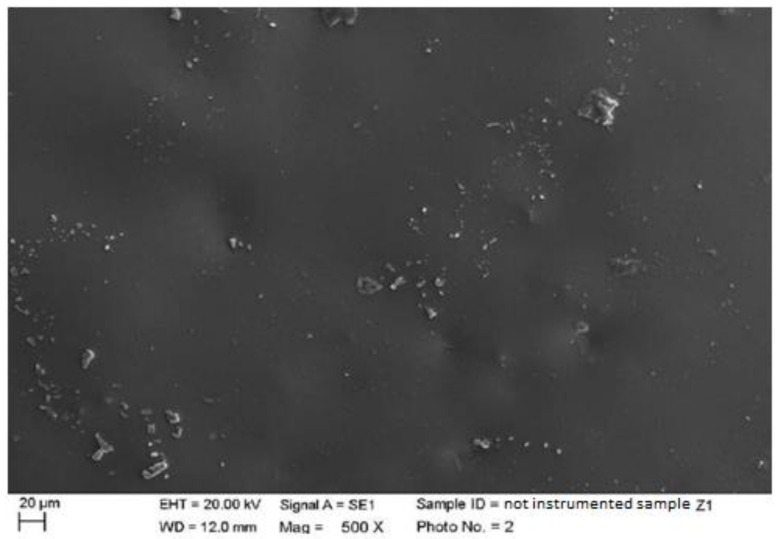
Non-instrumented zirconium sample Z1 (500×).

Zirconium, similar to lithium disilicate, had a surface with few processing residues of rather large dimensions. Similar to ceramic, on the other hand, it had a fracture area with clear edges caused by the finishing process of the element and determined by the greater hardness of this material (Figure 5).

### 4.1. SEM Analysis after Instrumentation

1. Composite

Instrumented composite with steel insert (PS, EMS, Nyon, Switzerland) mounted on Mini Piezon ultrasonic motor (EMS, Nyon, Switzerland). 

**Figure 6:**
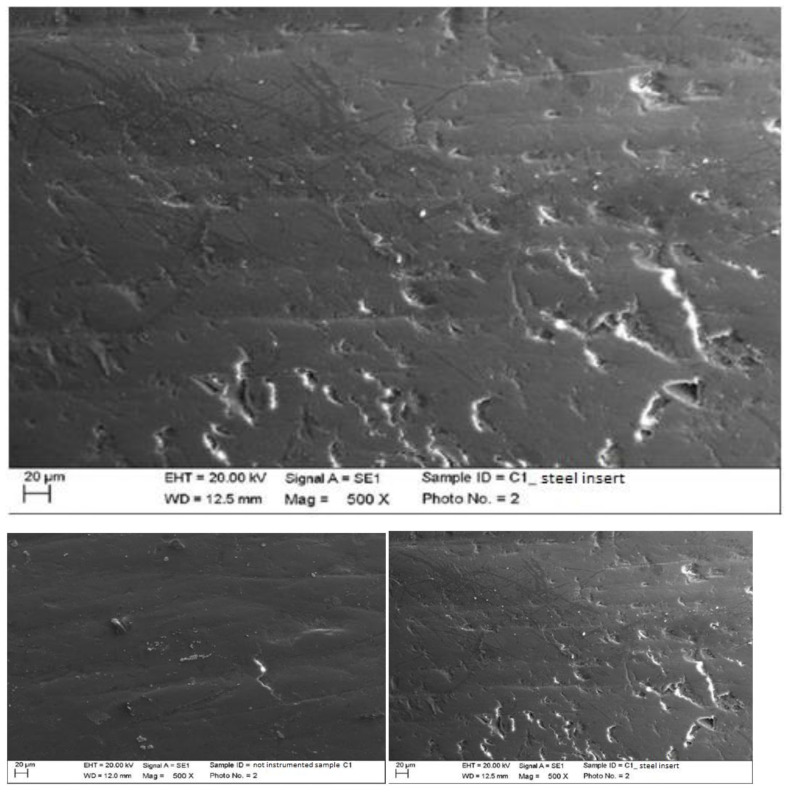
Instrumented composite sample C1 with steel insert (500×). Comparison before/after instrumentation.

The classic insert created light linear scratches on the surface without leaving accumulations of steel. The other irregularities were not due to the instrumentation, but to the finishing treatment of the material. Surface processing residues were removed from the treatment (Figure 6).

Instrumented composite with peek insert (ICS-IC1, Mectron, Carasco, Italy) mounted on MultiPiezo ultrasonic motor (Mectron, Carasco, Italy). 

**Figure 7:**
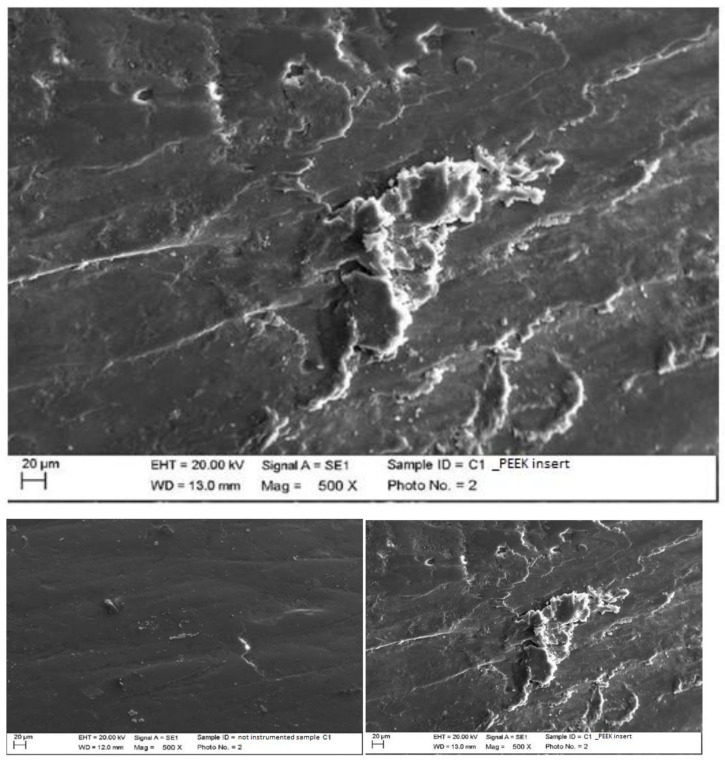
Instrumented composite sample C1 with peek insert (500×). Comparison before/after instrumentation.

The peek on the composite material created important alterations by partially removing processing residues. The smaller residues were completely removed, but some surface irregularities remained. However, with the simple surface analysis, we could not define whether the alterations were due to etching or the deposition of the instrumentation material (Figure 7).

Instrumented composite with steel curette (PDT, Missoula, MT, USA). 

**Figure 8:**
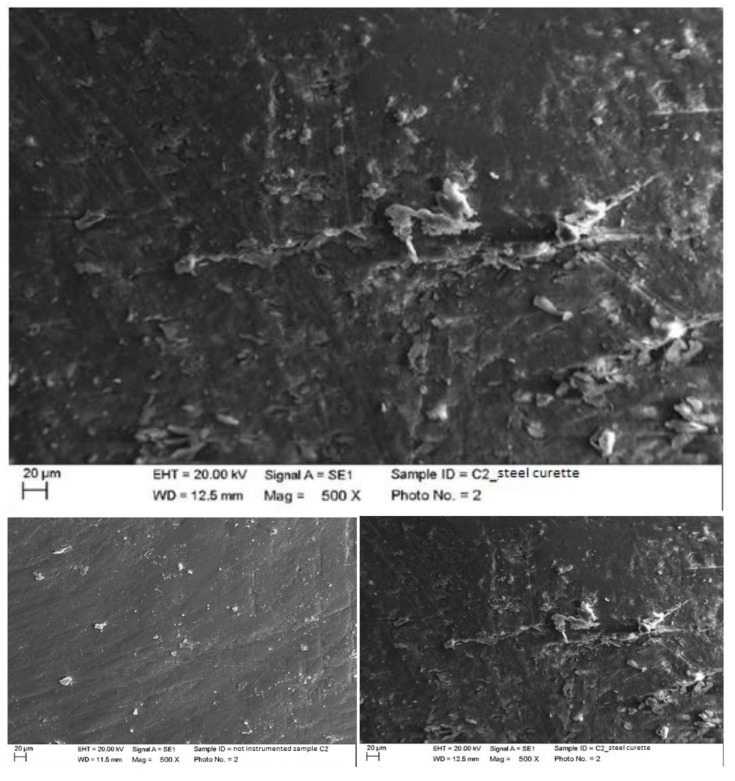
Instrumented composite sample C2 with steel curette (500×). Comparison before/after instrumentation.

The classic curette did not create important alterations to the surface of the material; it removed some residues, but left others (Figure 8).

Instrumented composite with titanium curette (Wingrove 3-4, PDT, Missoula, MT, USA).

**Figure 9:**
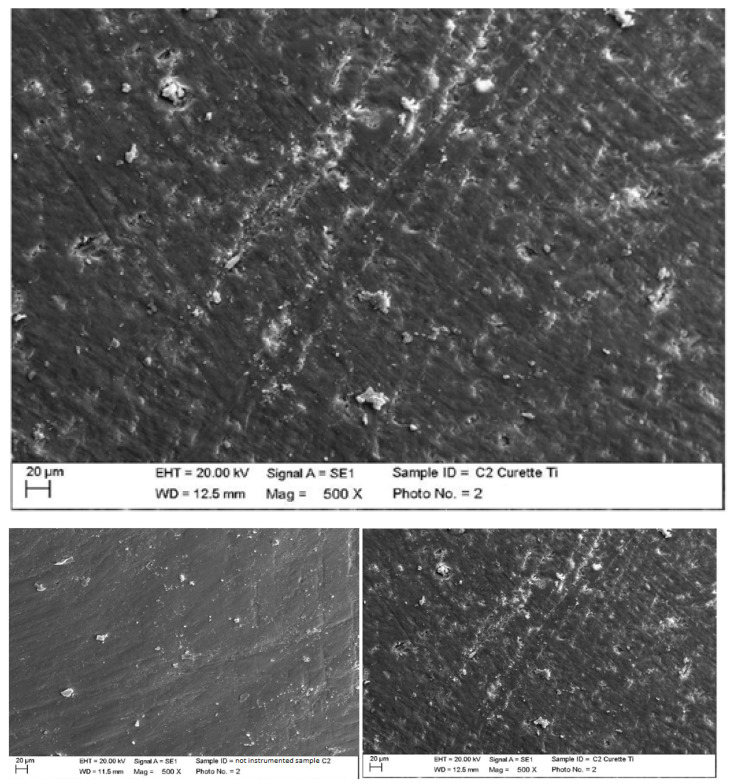
Instrumented composite sample C2 with titanium curette (500×). Comparison before/after instrumentation.

The titanium curette created deeper incisions than the classic curette, completely removing finishing residues (Figure 9).

2. Lithium disilicate

Instrumented disilicate with classic steel insert (PS, EMS, Nyon, Switzerland) mounted on Mini Piezon ultrasonic motor (EMS, Nyon, Switzerland).

**Figure 10:**
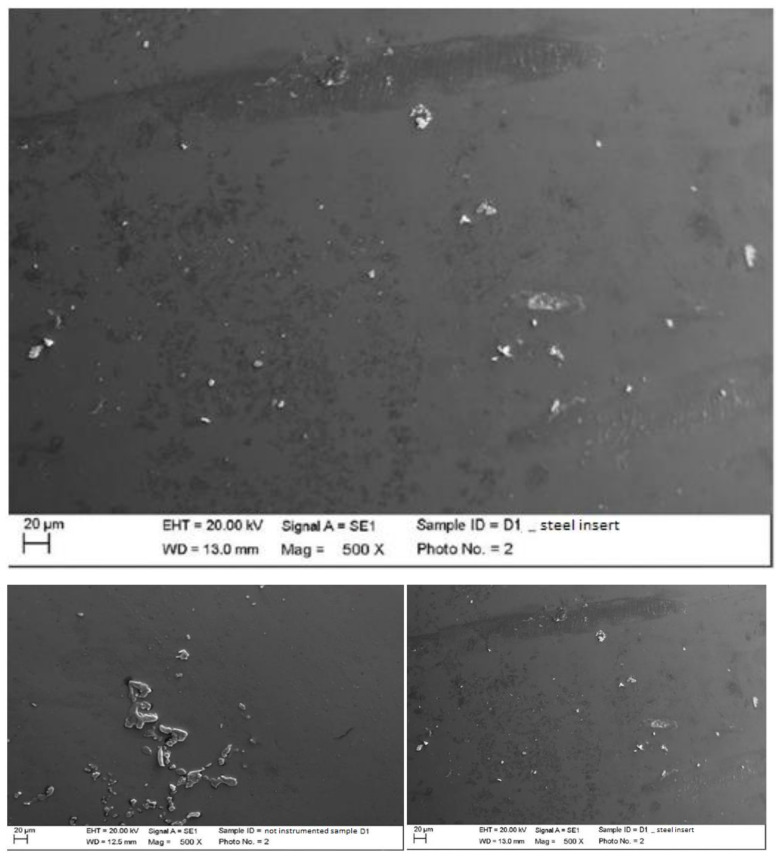
Instrumented disilicate sample D1 with steel insert (500×). Comparison before/after instrumentation.

The classic insert did not particularly alter the disilicate, also leaving processing residues, which was probably due to the fact that the residues themselves were larger than the other materials. There were slight scratches on the surface visible only at large magnifications (Figure 10).

**Figure 11:**
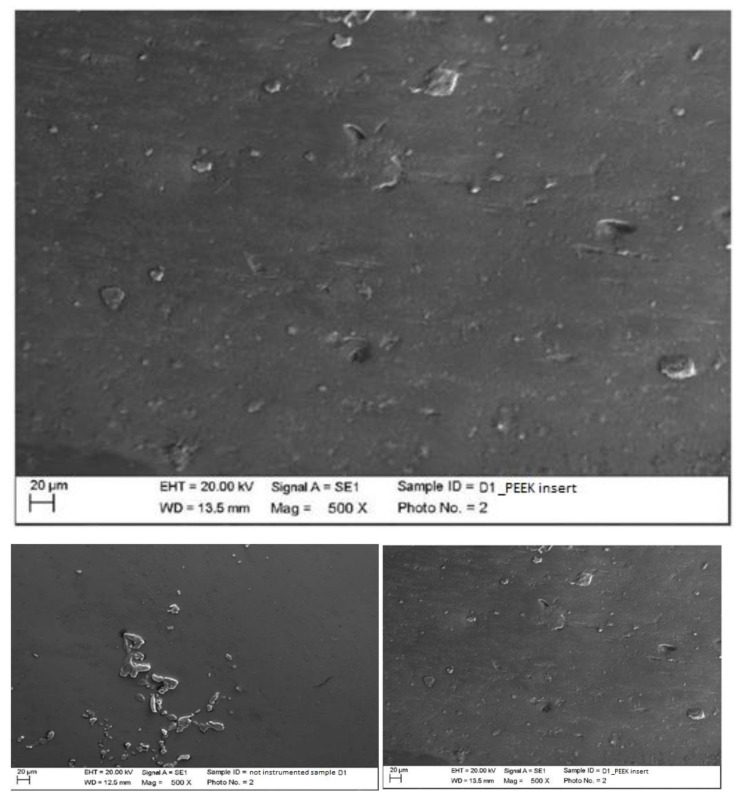
Instrumented disilicate sample D1 with peek insert (500×). Comparison before/after instrumentation.

The peek created major alterations on the surface of the lithium disilicate. In fact, the dendritic formations (or primary crystals) representing the origin of the material were clearly visible, exposed precisely following the instrumentation of the surface itself. However, it was not possible with surface analysis to establish whether the formations were abraded disilicate or residual peek; to verify this, chemical–physical analysis of the surface should be carried out. A photo was taken at 3800 magnification, where it is possible to see in detail the areas from which the crystals were formed (Figure 11).

Instrumented disilicate with steel curette (PDT, Missoula, MT, USA).

**Figure 12:**
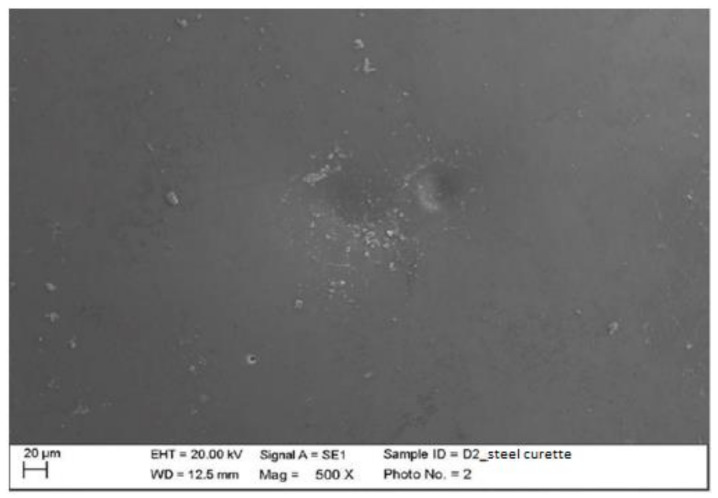
Instrumented disilicate sample D2 with steel curette (500×).

The classic curette created almost no effect on the disilicate, other than the slight accumulation of the instrumentation materials (Figure 12).

Instrumented disilicate with titanium curette (Wingrove 3-4, PDT, Missoula, MT, USA).

**Figure 13:**
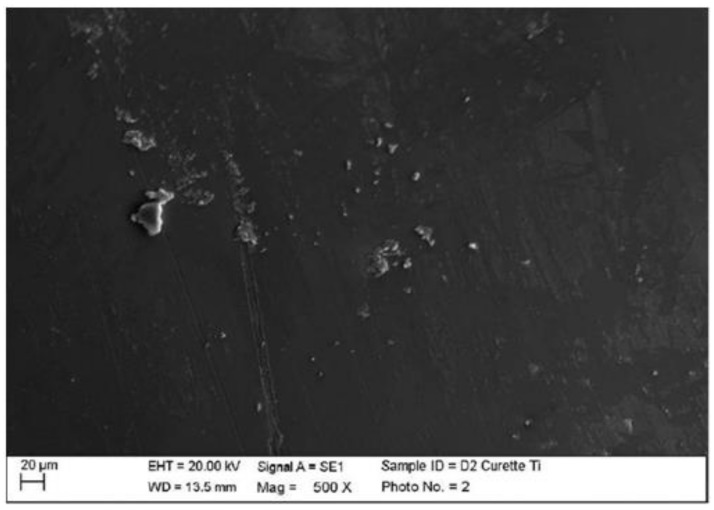
Instrumented disilicate sample D2 with titanium curette (500×).

The titanium curette created important and deep surface incisions while leaving instrumentation residues embedded within the grooves, which could be partly titanium pieces and partly pieces of silicone residues (Figure 13).

3. Ceramic

Instrumented ceramic with steel insert (PS, EMS, Nyon, Switzerland) mounted on Mini Piezon ultrasonic motor (EMS, Nyon, Switzerland). 

**Figure 14:**
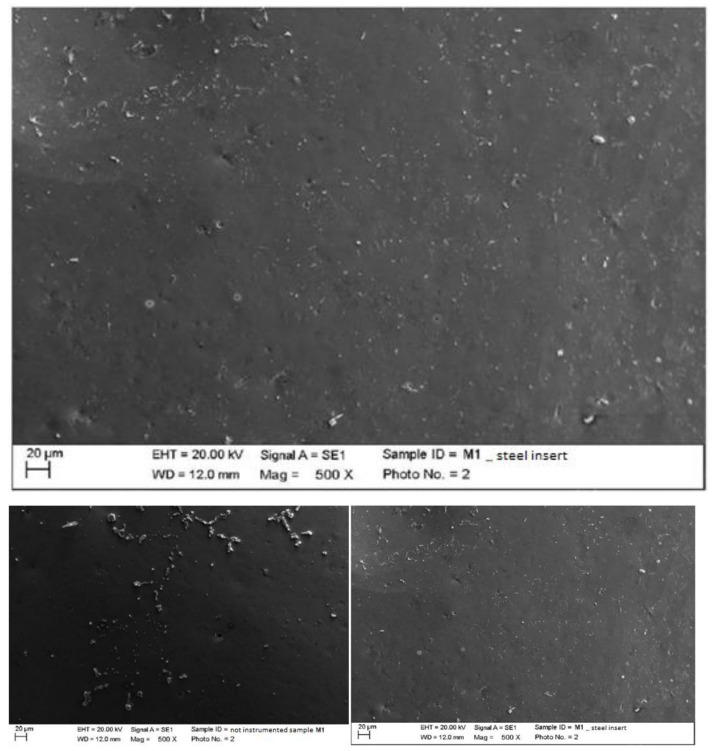
Instrumented ceramic sample M1 with steel insert (500×). Comparison before/after instrumentation.

The classic insert eliminated finishing residues by replacing them with other instrumentation residues. The elimination of residues was clearly visible at the higher magnification, which was comparable to a thin flaking film (Figure 14).

Instrumented ceramic with peek insert (ICS-IC1, Mectron, Carasco, Italy) mounted on ultrasonic motor, MultiPiezo (Mectron, Carasco, Italy).

**Figure 15:**
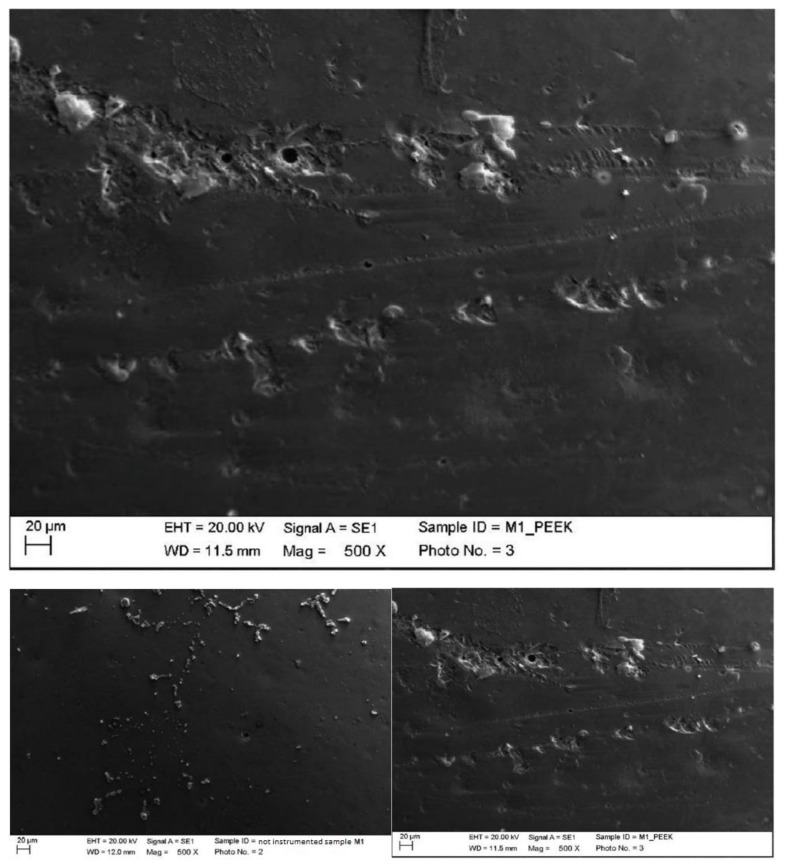
Instrumented ceramic sample M1 with peek insert (500×). Comparison before/after instrumentation.

The peek on the ceramic created horizontal and vertical alterations, but it was again not possible to establish the origin of these changes (Figure 15).

Instrumented ceramic with steel curette (PDT, Missoula, MT, USA). 

**Figure 16:**
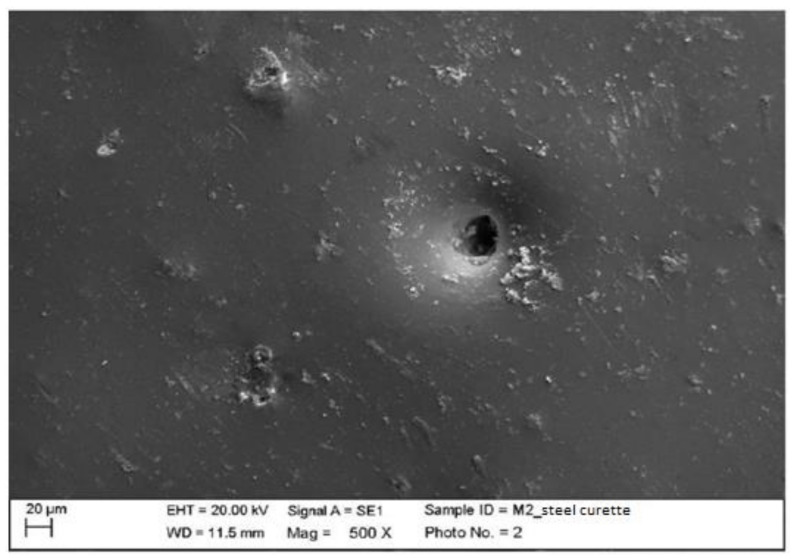
Instrumented ceramic sample M2 with steel curette (500×).

The classic curette, being ceramic, a very hard material, hardly created any negative effects on the surface. The crater visible in photo X was due to the ceramic material itself, which was hard and brittle, but was not related to manual instrumentation (Figure 16).

Instrumented ceramic with titanium curette (Wingrove 3-4, PDT, Missoula, MT, USA).

**Figure 17:**
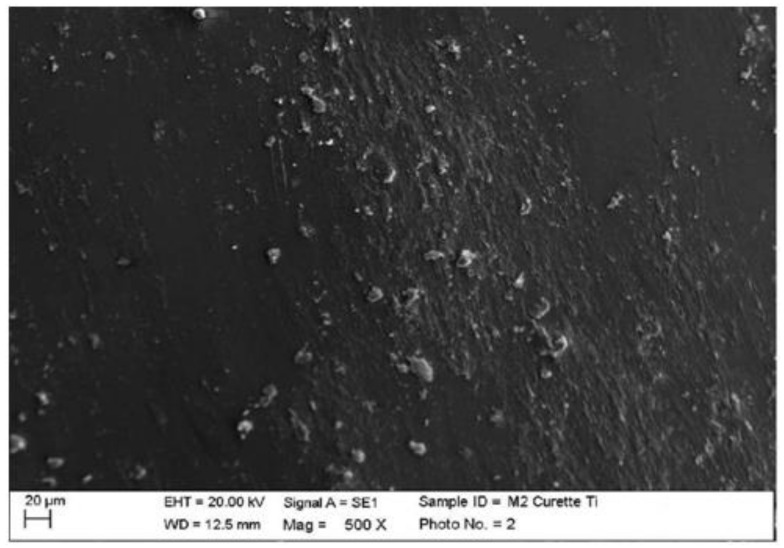
Instrumented ceramic sample M2 with titanium curette (500×).

The titanium curette did not create particular damage but left numerous large residues, both ceramic and titanium (Figure 17).

4. Zirconium

Instrumented zirconium with steel insert (PS, EMS, Nyon, Switzerland) mounted on Mini Piezon ultrasonic motor (EMS, Nyon, Switzerland).

**Figure 18:**
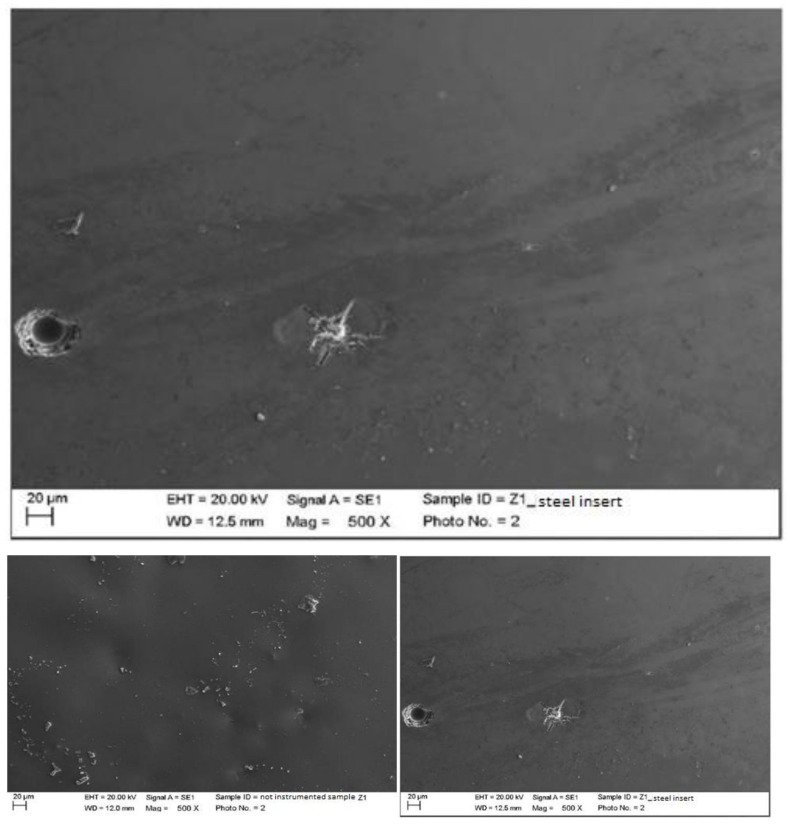
Instrumented zirconium sample Z1 with steel insert (500×). Comparison before/after instrumentation.

The classic insert created a large fracture, clearly visible at 2000 magnification, in an area that was previously irregular. The crater visible in image 5.42 was not due to the instrumentation, but, as seen previously in the ceramic, to the hardness/fragility of the material (material defect). The trimming residues were removed (Figure 18).

Instrumented zirconium with peek insert (ICS-IC1, Mectron, Carasco, Italy) mounted on ultrasonic motor, MultiPiezo (Mectron, Carasco, Italy). 

**Figure 19:**
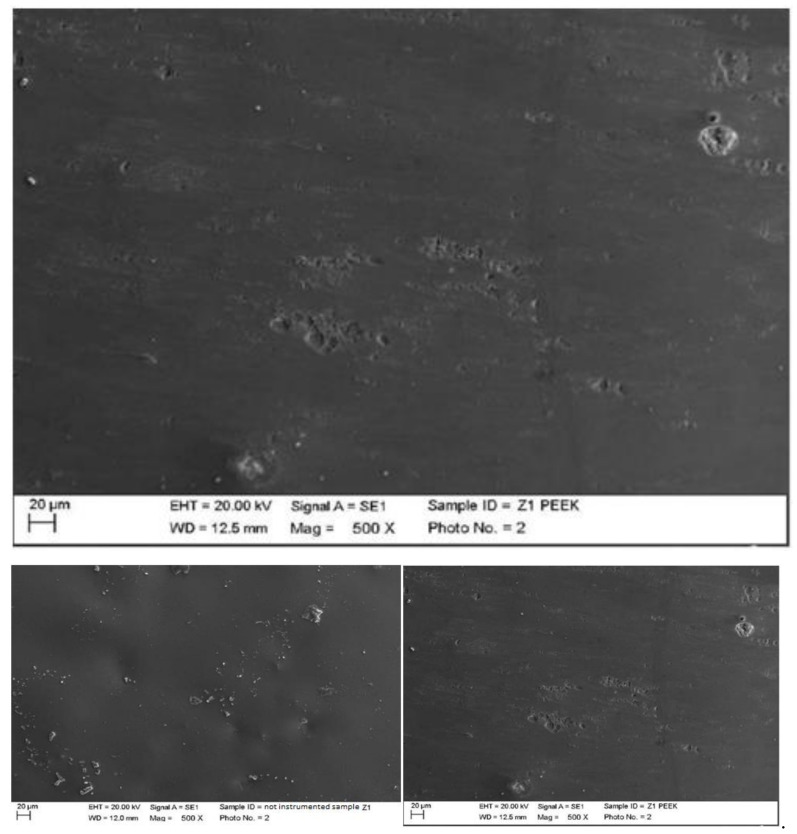
Instrumented zirconium sample Z1 with peek insert (500×). Comparison before/after instrumentation.

The peek created alterations on the zirconium; however, it was not possible to establish the cause. The instrumentation areas were clearly visible (Figure 19).

Instrumented zirconium with steel curette (PDT, Missoula, MT, USA). 

**Figure 20:**
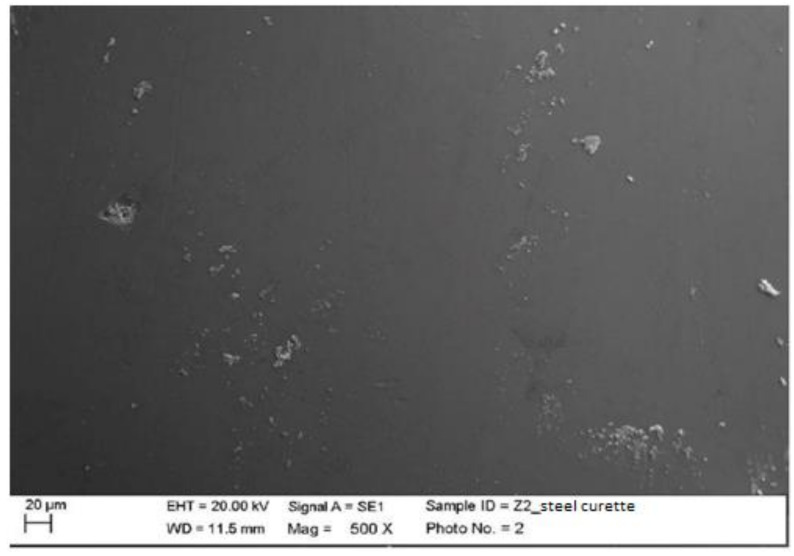
Instrumented zirconium sample Z2 with steel curette (500×).

At maximum magnification, incisions and surface residues were visible on the zirconium part treated with a steel curette (Figure 20).

Instrumented zirconium with titanium curette (Wingrove 3-4, PDT, Missoula, MT, USA).

**Figure 21:**
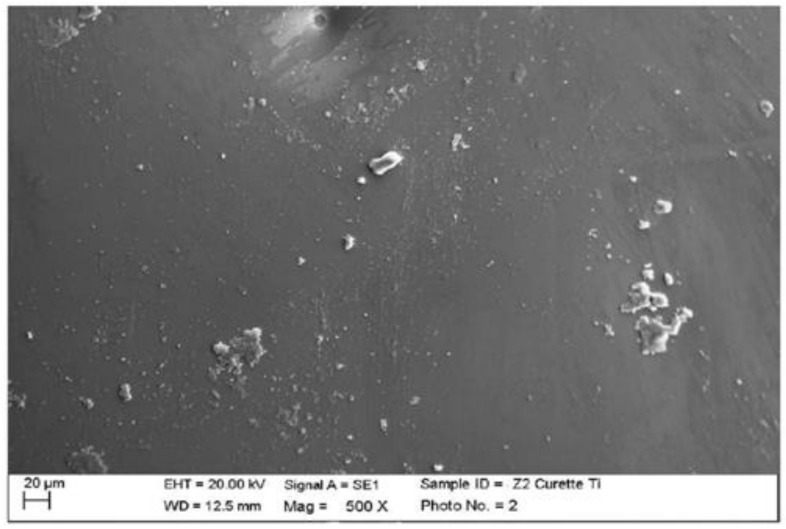
Instrumented zirconium sample Z2 with titanium curette (500×).

The titanium curette created slight superficial incisions visible at higher magnifications, leaving few processing residues. On the left side of the enlargement, 2000 super-surface residues from finishing were still visible (Figure 21).

### 4.2. Statistical Results

Two Kruskal -Wallis tests were performed in the statistical analysis of the roughness values.

**Figure 22:**
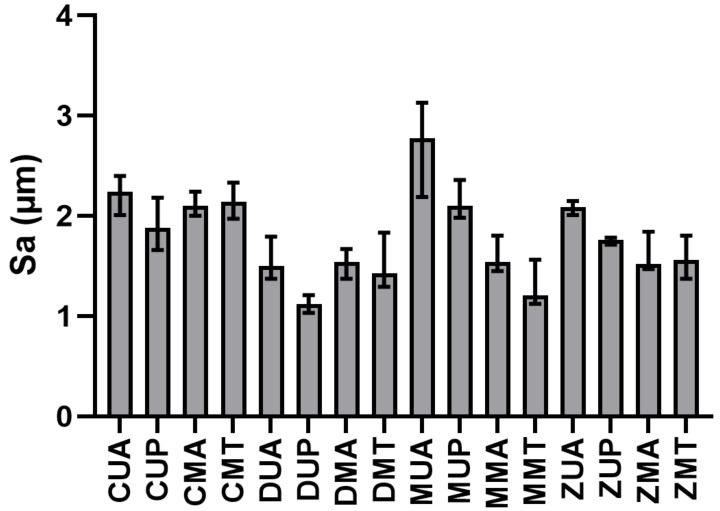
Median and extreme values of Sa for each combination. The material (C: composite, D: lithium disilicate, M: metal ceramic, Z: zirconium ceramic) and instrumentation (UA: ultrasonic-steel, UP: ultrasonic-peek, MA: manual-steel, MT: manual-titanium) are indicated along the abscissa axis.

In both cases, the 80 tests (five measurements × four materials × four instruments) were divided into four groups: composite, lithium disilicate, metal ceramic, and zirconium ceramic for material analysis, and ultrasonic-steel, ultrasonic-peek, manual-steel, and manual-titanium for instrumentation analysis. The null hypothesis, i.e., that all groups of samples came from the same distribution, was rejected in both tests, confirming the significance of the influence of both the material and the instrumentation (*p* < 0.05). The bar diagrams representing the median, maximum, and minimum values of Sa for each combination of material and instrumentation are shown in Figure 22, the legend of which is shown in Table 1.

The Kruskal-Wallis test confirmed that not all groups were from the same population, but did not provide information about which groups differed from each other. To determine between which materials and equipment there was a significant difference, a Bonferroni post hoc test was performed (Table 2 and Table 3). *p*-values less than 0.05 corresponded to pairs of materials and instruments that had a significant difference.

## 5. Discussion

From the analysis of the SEM images, it is possible to draw considerations on which instruments to use in relation to the prosthetic material. The classic steel insert mechanically eliminated finishing residues on all materials. The fact that the processing residues, small or large, were removed from the surface of the element, did not create particular problems as the residue itself did not affect the chemical-physical characteristics of the material. It could possibly be a positive aspect, considering that surfaces that are not smooth and with residues favor the accumulation of other substances present within the oral cavity [16,17,18,19]. Surface incisions were practically negligible on all materials, except for zirconium, where the insert created an important fracture in an area that, however, already had an initial irregularity. From this, we can understand how important careful polishing and finishing of the prosthesis is [20,21]. The peek insert used mechanically created alterations on all tested surfaces. In the case of disilicate, dendritic formations and crystals were visible. However, with the simple SEM surface analysis, we were not able to establish whether this created negative effects, as the changes could be due to the abrasion of the material or, more easily, to a deposit of plastic material that makes up the component. It follows that a chemical analysis of the aesthetic surfaces treated with peek could be the subject of a subsequent study. The steel curette, on the other hand, did not create particular problems, other than an increase in instrumentation residues, sometimes accompanied by a reduction in re-dyeing residues, with the exception of zirconium, where, at maximum magnification, it is possible to observe some scratch lines. Instrumentation with a titanium curette created deeper incisions than the steel curette in the composite and disilicate. Engravings were present, but lighter, on the surfaces of the ceramic and zirconium. In all cases, it left residues that may be partly due to the instrument itself and partly due to the scratching, whether greater or lesser, of the material under examination. The statistical analysis of the roughness supports the qualitative conclusions of the SEM analysis. In particular, the difference in terms of roughness between the steel ultrasound and manual curettes in both the titanium and steel is significant. This can support the thesis that mechanical instrumentation is less influenced by the operator’s manual skills. Among the materials, the composite showed the greatest surface roughness on all the instruments tested, followed by metal ceramic, zirconia, and disilicate. The realization of monolithic restorations (zirconia and disilicate) starting from industrially produced blocks likely gives the materials mechanical characteristics that make them less susceptible to damage from instrumentation than layered restorations. Further studies are needed to deepen this observation and analyze more instruments and prosthesis surfaces [22,23].

## 6. Conclusions

In conclusion, from the analysis performed, we can state that peek inserts are the least aggressive for the treatment of aesthetic prosthetic surfaces. However, the data on the effectiveness of peek ultrasounds in the literature are conflicting, so, for the maintenance of the surfaces over time, the best choice seems to be mechanical treatment with a steel insert.

The material less susceptible over time to professional hygiene maneuvers was lithium disilicate followed by zirconia and metal ceramic; with reference to this aspect, the worst results were attributable to the composite.

## Figures and Tables

**Table 1:** Legend of graphic labels.

Instrumentation Performed	Label Table 1
Instrumented composite with ultrasonic steel insert lingual surface first elements	CUA
Instrumented composite with ultrasonic peek insert vestibular surface first elements	CUP
Instrumented composite with steel curette lingual surfaces of the second elements	CMA
Instrumented composite with titanium insert vestibular surfaces of the second elements	CMT
Instrumented disilicate with ultrasonic steel insert lingual surface first elements	DUA
Instrumented disilicate with ultrasonic peek insert vestibular surface first elements	DUP
Instrumented disilicate with steel curette lingual surfaces of the second elements	DMA
Instrumented disilicate with titanium insert vestibular surfaces of the second elements	DMT
Instrumented ceramic with ultrasonic steel insert lingual surface first elements	MUA
Instrumented ceramic with ultrasonic peek insert vestibular surface first elements	MUP
Instrumented ceramic with steel curette lingual surfaces of the second elements	MMA
Instrumented ceramic with titanium insert vestibular surfaces of the second elements	MMT
Instrumented zirconia with ultrasonic steel insert lingual surface first elements	ZUA
Instrumented zirconia with ultrasonic peek insert vestibular surface first elements	ZUP
Instrumented zirconia with steel curette lingual surfaces of the second elements	ZMA
Instrumented zirconia with titanium insert vestibular surfaces of the second elements	ZMT

**Table 2:** Bonferroni test—Materials.

Material		*p* (Bonferroni)
Composite	Lithium disilicate	1.47 × 10^−7^
Composite	Metal ceramic	0.235
Composite	Zirconia ceramic	0.023
Lithium disilicate	Metal ceramic	0.003
Lithium disilicate	Zirconia ceramic	0.054
Metal ceramic	Zirconia ceramic	1

**Table 3:** Bonferroni test—Instrumentation.

Instrumentation		*p* (Bonferroni)
Ultrasonic-steel	Ultrasonic-peek	0.089
Ultrasonic-steel	Manual-steel	0.032
Ultrasonic-steel	Manual-titanium	0.002
Ultrasonic-peek	Manual-steel	1
Ultrasonic-peek	Manual-titanium	1
Manual-steel	Manual-titanium	1

## Data Availability

The data presented in this study are available on request from the corresponding author.
